# Self- and Parent-Reported Psychological Symptoms in Young Cancer Survivors and Control Peers: Results from a Clinical Center

**DOI:** 10.3390/jcm9113444

**Published:** 2020-10-27

**Authors:** Rita Barone, Mariangela Gulisano, Emanuela Cannata, Sara Padalino, Federica Saia, Nicoletta Maugeri, Fabio Pettinato, Luca Lo Nigro, Antonino Casabona, Giovanna Russo, Andrea Di Cataldo, Renata Rizzo

**Affiliations:** 1 Child Neuropsychiatry Unit, Department of Clinical and Experimental Medicine, School of Medicine, University of Catania, 95123 Catania, Italy; mariangelagulisano@gmail.com (M.G.); sara.padalino@hotmail.it (S.P.); federicasaia@live.com (F.S.); nico_maugeri@hotmail.it (N.M.); fabiopettinato91@gmail.com (F.P.); rerizzo@unict.it (R.R.); 2 Pediatric Oncohematology Unit, Department of Clinical and Experimental Medicine, School of Medicine, University of Catania, 95123 Catania, Italy; e.cannata80@gmail.com (E.C.); lonigro@policlinico.unict.it (L.L.N.); diberuss@unict.it (G.R.); adicata@unict.it (A.D.C.); 3 Department of Biomedical and Biotechnological Sciences, Section of Physiology, School of Medicine, University of Catania, 95123 Catania, Italy; casabona@unict.it

**Keywords:** pediatric cancer survivors, emotional symptoms, self-reported symptoms, parent-reported symptoms, symptoms co-occurrence

## Abstract

Pediatric cancer survivors are at increased risk for psychological distress. We sought to understand the severity and symptoms’ co-occurrence among pediatric survivors compared to controls by rating both self- and parent-reported symptomatology. Forty survivors (22 males; mean age at study time: 12.9 years) participated in the study. Most survivors (85%) had a diagnosis of acute lymphoblastic leukemia. Seventy-nine healthy controls with the same age and gender distribution as the patients were included. A standardized assessment of psychological functioning was conducted by self- and parent-reported symptoms evaluations. The self-reported anxious symptom severity was significantly higher in survivors. A significantly higher proportion of survivors compared to controls had clinically significant anxiety, depression, and combined anxiety symptoms (i.e., social anxiety, separation anxiety, or physical symptoms). In both study groups, the self-reported emotional and somatic symptoms were significantly associated. The multi-informant assessments of the psychological symptoms revealed distinct associations between the child- and parent-reported symptoms in the survivors’ group: the survivors’ self-reports of depressive symptoms, somatic symptoms, and functional impairment were significantly correlated with the parent reports of child behavioral concerns, somatic complaints, and functional impairment, respectively. Conclusion: Self-reported symptoms showed similar comorbidity profiles in survivors and control peers. The multi-informant assessments detected differences in the association of self- and parent-reported symptoms between the survivor and control groups. The present study showed that multi-informant assessment is critical to understanding symptom profiles and to informing intervention with particular regard to parental participation and support.

## 1. Introduction

Pediatric cancer survivors are ultimately at risk of neurocognitive and psychological late effects of their disease [1,2,3,4]. The most well established neurocognitive deficits include attention, verbal memory, working memory, and processing speed [5]. Although there is considerable variability in the individual outcomes, almost 35% of all pediatric survivors have some difficulties in learning skills [6]. Compared to consistent results on neurocognitive profiles and academic achievements, the data on psychological functioning are more controversial. Previous studies showed that in general, pediatric cancer survivors do not necessarily complain about psychological symptoms. However, emotional symptoms, behavioral alterations, somatization, and lifetime post-traumatic distress symptoms have been reported. [2,3,7]. In general, psychological distress and emotional symptoms were observed in 13 to 29% of adolescent survivors. Anxiety disorders and depression were rated in 7% and 6% when specifically measured [8]. The severity of anxiety and somatization symptoms in adolescent survivors (13%) exceeded that of their siblings with psychological distress (11%) and psychotherapy patients [7]. Adult survivors of pediatric cancer showed higher frequencies of affective, somatic, and comorbid symptoms (38%) than their siblings (26%) serving as controls. In particular, distinct subsets were detected based on psychological symptoms, such as asymptomatic subjects, individuals with isolated affective distress, or somatic symptoms. Of note, emotional and somatic symptoms in comorbidity were found in almost 11% of adult survivors [9]. Results from the Childhood Cancer Survivor Study confirmed that a variable proportion of young survivors (aged 12 to 17 years; median time since diagnosis = 12.4 years) experienced the co-occurrence of emotional, behavioral, and social symptoms that were associated with treatment late effects [10]. In particular, among patients not exposed to cranial radiotherapy (CRT), three symptom profiles were identified: (a) survivors with greater internalizing of emotional symptoms, social withdrawal, and attention problems (16.3%); (b) survivors with greater symptoms of externalizing behavior and an attention deficit (8.8%); (c) survivors with increased symptoms across all domains (5.3%) [10]. Based on the abovementioned findings, exploring the comorbid symptomatology in survivors deserves interest. In this regard, recent research has highlighted the importance of multi-informant assessment to evaluate psychological symptoms in survivors [11,12]. Childhood acute lymphoblastic leukemia (ALL) is the most common pediatric cancer. Many children with ALL are diagnosed and treated before the age of 6, with improved overall five-year survival rates that are greater than 80% [1]. Among the survivors of ALL, parents reported their children to have more depression symptoms (16.5%), as well as multiple symptoms of internalizing problems (i.e., anxiety/depression: 17.1%), relative to the general population frequency [11]. Moreover, symptoms of inattention reported by survivors of ALL were consistent with the parent reports of survivor attention problems [12]. Limited information is available on self-reported functional impairment and parent-reported child functioning in cancer survivors relative to controls. Survivors were similar to the controls in self-reported functional impairment. In both groups, the self-reported and parent-reported functioning were found to be correlated [13]. In the present study, we investigated emotional, somatic, and behavioral symptoms in a clinical sample of pediatric cancer survivors in comparison with healthy control peers. The large majority of the studied survivors had childhood ALL, thus representing an almost homogeneous diagnostic sample. We sought to understand the severity of the symptoms and their co-occurrence among pediatric survivors relative to controls by rating both the self- and parent-reported symptomatology. Profiling survivors’ symptoms using multi-informant assessments is particularly important for defining outcomes and treatment, particularly at a younger age.

## 2. Materials and Methods

### 2.1. Participants

This cross-sectional cohort study was undertaken in the context of a local larger project dedicated to the long-term effects of pediatric cancer in young survivors. Ninety-five subjects who survived pediatric cancer were consecutively approached to participate during annual outpatient visits at the Pediatric Oncohematology Unit and evaluated at the Child Neuropsychiatry Unit University Hospital of Catania (Italy) during the study period (November 2018–January 2019). The inclusion criteria were as follows: (1) subjects aged between 8 and 16 who survived cancer diagnosed before the age of 10 and (2) the absence of diagnosed neurodevelopmental disturbances and systemic illness other than previous cancer. The exclusion criteria included: (1) disagreement with the neuropsychiatric evaluation, (2) the patient was diagnosed with a brain tumor, and (3) the presence of systemic illness or diagnosed neuropsychiatric diseases. As a control group, 79 subjects with the same age and gender distribution as the patients were recruited from healthy volunteer students attending primary and secondary schools in the city of Catania.

The protocol was approved by the internal Institutional Review Board (ethical approval code: 01/2018), after considering the heads of Pediatric Hemato-Oncology and Neuropsychiatry Units, plus all physicians and nurses of the same units. The procedures performed were in accordance with the principles of the 1964 Declaration of Helsinki and its later amendments (2013). Written informed consent was obtained from all study participants and their parents.

### 2.2. Standardized Assessment

The study was conducted by self- and parent-reported symptom evaluations. The cognitive level (full-scale intelligence quotient (FIQ)) was assessed using the Wechsler Intelligence Scale for Children (WISC) III edition or its related short form [14].

### 2.3. Anxiety and Depression Symptoms

Anxiety symptoms were assessed using the Multidimensional Anxiety Scale for Children (MASC), which is a 39-item questionnaire providing a self-report measure of anxiety in 8–19-year-olds. The MASC produces a total score, as well as scores for four subscales: physical symptoms, harm avoidance, social anxiety, and separation anxiety [15]. A MASC T-score of ≥65 was considered clinically significant. Depressive symptoms were assessed using the Children’s Depression Inventory (CDI), which is a self-reported measure of depressive symptoms designed for children from 7 to 17 years old. T-scores of ≥65 were classified as clinically significant [16].

### 2.4. Somatic Symptoms

Somatic symptoms were evaluated using the Children’s Somatic Symptoms Inventory 24 (CSSI-24; formerly known as the Children’s Somatization Inventory (CSI-24) [14]. The CSSI-24 is a child-reported (CSSI-C) and parent-reported (CSSI-P) dimensional measure of somatic symptoms on a five-point scale from 0 (not at all) to 4 (a whole lot). The total score from 0 to 96 is calculated by computing the sum of the item responses. Tertile-derived clinical reference points for the CSSI-24 total score (<18, low; 19–31, moderate; ≥32, high) were used [17].

### 2.5. Behavioral Symptoms

Behavioral patterns were screened using the Child Behavior Checklist, which is a well-established and widely used parent-completed measure of global, internalizing, and externalizing behavioral symptoms in children and adolescents aged 1.5–18 years. According to normative data, T-scores between 60 and 64 or ≥65 indicate that the subject is at risk or clinically affected by behavioral symptoms, respectively [18].

### 2.6. Functional Disability Measure

Child impairment was evaluated using the Functional Disability Inventory (FDI), which is a 15-item self-reported and parent-reported measure that assesses perceived difficulty in performing common activities in the domains of school, home, recreation, and social interactions. The FDI yields a total score that can range from 0 to 60, with higher scores indicating greater disability. Clinical reference points for the FDI total score were used (<12, low; 13–29, moderate; ≥30, high) [19].

### 2.7. Data Analysis

Data are presented as means and standard deviations (SDs) for continuous variables, and absolute frequencies and percentages for categorical variables. Student’s *t*-test was used to compare the mean values of the FIQ, MASC, CDI, CBCL T-scores, CSSI-24, and FDI raw scores between the study groups. Due to multiple comparisons, a Bonferroni adjustment was adopted to correct for type I error inflation. The alpha level that was considered after the Bonferroni correction was set to 0.007. The comparison of the proportions between groups was conducted using the chi-squared test with a Yates correction. Correlations between study variables were analyzed using Pearson correlation analysis. Multiple linear regression analysis was used to test whether each studied feature could be predicted by changes in disease-related characteristics, such as age at study, age at diagnosis, and age at ending therapy. A stepwise backward elimination procedure was performed in order to eliminate the non-significant covariates and to avoid any possible redundancy in the regression model due to correlations between predictors. This procedure started with a full model and removed the least significant predictor at each iteration until only significant variables remained. In the case of the final models including two covariates, we computed the standardized coefficients (SCs) to estimate the contribution of each variable to the total variance. Differences with *p* ≤ 0.05 were considered significant. Data were analyzed using SPSS software v. 23 (*SPS*, Bologna, Italy).

## 3. Results

**Figure 1:**
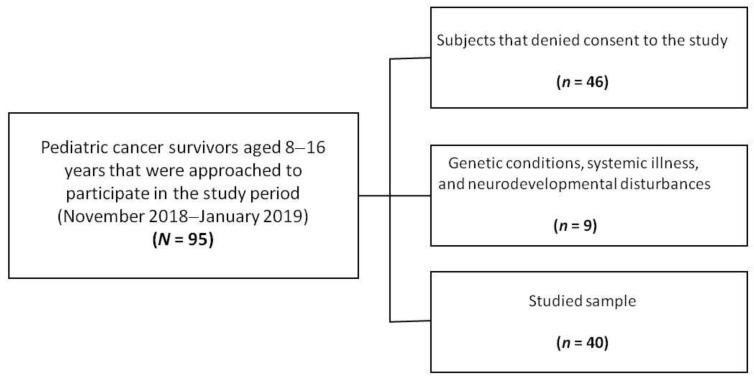
Diagram of the pediatric cancer survivor study participation.

The enrollment system for the current study is depicted in Figure 1. Of the 95 pediatric cancer survivors approached to participate, nine subjects (9%) were excluded owing to the presence of systemic, syndromic, or neurodevelopmental disturbances, such as trisomy 21 (*n* = 2), Kabuki syndrome (*n* = 2), autism spectrum disorder (*n* = 1), congenital deafness and developmental disability (*n* = 2), neurogenic bladder (*n* = 1), and maturity-onset diabetes of the young (MODY) (*n* = 1). Forty-six subjects (53%) refused to participate. In the end, 40 pediatric cancer survivors were enrolled (recruitment rate of 46%). They included 22 males (55%) and 18 females (45%) aged from 9 to 16 years (mean age = 12.9, SD = 3.1). The majority of patients had a diagnosis of ALL (*n* = 34 patients, 85%). Other diagnoses included Burkitt lymphoma (*n* = 2, 5%), Wilms’ tumor (*n* = 2, 5%), hepatoblastoma (*n* = 1, 2.5%), and rhabdoid tumor (*n* = 1, 2.5%). The age at diagnosis was 4.26 ± 2.81 years with an off-therapy period at the study time equal to 7.58 ± 3.35 years. The treatment plan included chemotherapy in all patients. Four subjects received radiation therapy. In particular, patients with ALL received a two-year program of standard chemotherapy according to AIEOP-BFM (Associazione Italiana di Ematologia e Oncologia Pediatrica & Berlin-Frankfurt-Münster) 2000 and 2009 protocols [20,21]. The patient with lymphoma received only chemotherapy, according to the AIEOP LNH (Associazione Italiana di Ematologia e Oncologia Pediatrica Linfoma non Hodgkin) 97 protocol [22]. Nephrectomy, chemotherapy, and radiotherapy as part of the AIEOP TW (Associazione Italiana di Ematologia e Oncologia Pediatrica Tumore di Wilms) 2003 protocol were administered to Wilms’ tumor patients [23]. The patient with a rhabdoid tumor received adjuvant chemotherapy for 30 weeks, combining vincristine, doxorubicin, cyclophosphamide, carboplatin, and etoposide [24].

The healthy controls comprised 46 males (58%) and 33 females (42%) with ages ranging from 9 to 16 years (mean 12.5, SD 3.3). The results of the cognitive evaluation and psychological functioning are reported in Table 1 and Table 2. The mean total IQ levels were not significantly different between the two groups (*p* = 0.357). Out of the 40 survivors, 31 (77%) underwent a cognitive assessment. Five (16%) had a full IQ < 85 (threshold value of normal intellectual functioning). In particular, three children had a borderline cognitive functioning on WISC III, whereas two subjects showed a full IQ of 68 and 65, respectively, which is symptomatic of mild intellectual disability. As for the control group, 50 of the 79 children (63%) underwent cognitive assessment using the WISC III short form. Five children (10%) showed borderline cognitive functioning. None had a total IQ symptomatic of intellectual disability.

### 3.1. Emotional Distress Symptoms

The total MASC T-scores were significantly higher in the survivor group (M = 48.3, SD = 10.89) than in the control subjects (M = 42.96, SD = 10.22, *t*(116) = 3.24, *p* = 0.0016), indicating that the self-reported anxious symptom levels were more severe in the cancer survivors. Regarding the depressive symptoms, no significant differences in the total CDI T-scores were observed between the two groups (Table 1).

### 3.2. Behavioral Symptoms

The behavioral patterns were screened using the Child Behavior Checklist (CBCL), which was completed by all the participants’ parents. A clinical cut-off point of ≥65 was considered. On average, the total CBCL T-scores were higher among survivors (M = 47.08, SD = 9.82) than their healthy control peers (M = 43.01, SD = 11.02, *t*(117) = 2.43, *p* = 0.016) (Table 1). Although not significant after the Bonferroni correction, the difference suggested that young survivors might be prone to developing more behavioral problems. Two survivors (5%) and three controls (3.7%) showed CBCL total T-scores in the clinical range (≥65) (*p* = 0.861). No significant differences were observed in the number of participants with internalizing or externalizing behavioral symptoms above the clinical cut-off between the two groups.

### 3.3. Somatic Symptoms

The somatic symptoms were measured using the Children’s Somatic Symptoms Inventory 24 (CSSI-24). On average, no significant differences in the severity of the somatic symptoms (CSSI-24 child and parent forms) were observed between the two groups (Table 1). Regarding the survivors, 35 out of 40 completed the questionnaire. Most of them (*n* = 31, 88%) had total scores between 0 and 18 (low somatic symptoms), whereas 4 (11%) had total scores in the moderate or high somatic symptom range. Likewise, out of the 79 control subjects, 75 (94%) presented total scores on the low somatic symptom range; 10 (12.6%) had moderate somatic symptom levels. The CSSI-24 parent form was administered to all participants’ parents and the total scores were between 0 and 18 (lowest tertile, low risk).

### 3.4. Functional Disability

Child impairment was evaluated using the Functional Disability Inventory (FDI). Thirty-five survivors (87.5%) and 100% of the controls completed the questionnaire. In total, no significant differences between the groups were observed for the occurrence of child-reported functional impairment. Among the survivors, the large majority (95%) had total scores in the low disability range, while 2 subjects (5%) were in the moderate range of functional disability (scores between 13 and 29). Likewise, among the controls, most (94%) were in the low range of functional disability. Five (6.3%) scored between 13 and 29, indicating moderate functional disability in these subjects. The results from the FDI-parent forms were also consistent with a low risk for functional disability in both groups (scores between 0 and 12).

### 3.5. Symptoms Severity and Co-occurrence

For analytical purposes, the participants who scored within the at-risk or clinically significant range were combined into a single “concerning range.” The proportion of subjects in the concerning range in one or more MASC anxiety syndrome subscales was significantly higher in the survivor group (*n* = 14, 36.8%) than in the control group (*n* = 10, 12.9%) (*p* = 0.008). Similarly to anxiety symptoms, the proportion of survivors with clinically significant scores for depression was significantly higher than that of the control subjects (*n* = 6, 15% vs. *n* = 4, 5%) (*p* = 0.023). Taken together, we found that a higher proportion of participants in the survivor group had emotional distress, which was defined by the presence of anxiety and depression symptoms in the concerning range. Regarding the co-occurrence of symptoms, we evaluated the possible associations between the psychological symptoms in all participants (Table 2). Moreover, we assessed whether the psychological symptoms could affect the overall functioning as measured by the FDI. We found that in both the survivor group and the control group, the self-reported emotional symptoms and the self-reported somatic complaints were significantly associated (*p* < 0.01), representing a shared comorbidity pattern between the groups. Likewise, the self-reported somatic symptoms were associated with self-reported functional impairment in both study groups (*p* = 0.0001). On the other hand, the self- and parent-reported symptoms analyses revealed some distinct associations in the survivors’ group: the survivors’ self-reporting of depressive symptoms, somatic symptoms, and functional impairment were significantly correlated with the parent-reporting of child behavioral concerns, somatic complaints, and functional impairment, respectively, with all pairwise Pearson correlation coefficients ranging from 0.37 to 0.79 and all *p*-values < 0.005 (Table 2). In contrast, in the control group, the parent-reporting of child behavioral problems was associated with the parent-reporting of child functional impairment (*p* = 0.0002). No significant associations between total IQ at study time and psychological symptoms were found. As a whole, the current data suggested that survivors’ self-reported depressive and somatic symptoms were well perceived by parents as somatic complaints, anxious/depressed behavior, and functional impairment in their children.

### 3.6. Multivariate Analysis of the Clinical Parameters and Age-Related Variables

We investigated whether the measured psychological symptoms were influenced by the age at study, age at diagnosis, and age at ending therapy in all survivors and in the survivors’ subgroup with emotional distress. A stepwise backward regression analysis revealed that there were significant changes only for some clinical parameters when the three age-related variables were used as predictors. In the entire survivors’ group, the combination of age at diagnosis and age at ending therapy produced the best regression model for the CSSI-24 child total (*r* = 0.59, *p* = 0.002, SC: −0.83 for age at diagnosis and 1.02 for age at ending therapy) and for the FDI child total (*r* = 0.62, *p* = 0.001, SC: −1.02 for age at diagnosis and 1.02 for age at ending therapy). In the subgroup of patients with emotional distress, the age at diagnosis was the best predictor for the MASC total T-score (*r* = 0.62, *p* = 0.01, SC: −0.62). Moreover, the combination of the age at diagnosis and age at ending therapy produced the best regression model for the CSSI-24 child total (*r* = 0.68, *p* = 0.001, SC: −0.66 for age at diagnosis and 0.77 for age at ending therapy) in survivors with emotional distress. Taken together, the multivariate analyses highlighted that some parameters delineating the disease duration had an influence on the occurrence of somatic symptoms in survivors. Moreover, earlier disease experience may have impacted the anxiety levels in survivors with emotional distress.

## 4. Discussion

The present study was undertaken to evaluate the self- and parent-reported emotional, somatic, and behavioral symptoms in a clinical sample of young cancer survivors compared to a healthy control group. Most study survivors had ALL diagnoses, thus representing a nearly homogeneous diagnostic sample. The severity of symptoms and their co-occurrence in the same individual were considered. We found that, on average, the children treated for cancer were doing well psychologically. However, the self-reported anxious symptom severity (MASC total T-scores) was significantly higher in cancer survivors. A higher proportion of young survivors compared to the controls had emotional distress, which was defined by the presence of symptoms that scored in the at-risk or clinically significant range for anxiety (36.8% vs. 12.9%) and depression (15% vs. 5%). It is worthy of note that participants with co-occurrence of multiple clinically significant anxiety symptoms (i.e., social anxiety, separation anxiety, or physical symptoms) were significantly more represented in the survivor group (12.5%) than in the control group (1.2%).

The present study adds to a growing body of literature that highlights that a minor but significant proportion of young survivors ranging from 20% to approximately 40% experience emotional concerns and psychological difficulties [7,8,10]. Variability in the prevalence rates of psychological symptoms among pediatric survivors reflects some inconsistencies that could depend on the characteristics of the study, such as differences in the type of assessment tools and informants involved, as well as differences in the characteristics of the survivors. The latter include different cancer diagnostic categories and treatments, as well as diversity in the considered ages at study time, in the time since diagnosis, and in the time since treatment completion. Moreover, the sample size and the presence and characteristics of control groups are additional variables among pertinent studies [8,25]. In addition to the abovementioned factors, relevant sample bias(es) might have an influence on the prevalence rates of psychological symptoms in young cancer survivors [26]. In particular, families of children with greater concerns might well have chosen not to participate when asking to focus on cancer-related family distress. In this regard, almost half of families (*n* = 46, 53%) initially recruited for the present study denied their consent because of different complaints. In particular, the long distance from the clinic (*n* = 10, 21%), familial appointments (*n* = 8, 17%), and unwillingness to participate (*n* = 38, 82%) were the main reasons for refusal. This high rate of unwillingness to join the study may suggest low feasibility and acceptability with our tools. However, it might also be that families with a lower level of adjustment wished to avoid confronting the difficulties experienced by themselves and their children. In the end, the high rate of families that denied their participation suggests that a proportion of young survivors are at risk for not pursuing long-term survivorship care. Likewise, similar recruitment rates were recently reported in pediatric cancer patients in on-therapy (56.6%) and off-therapy (47.3%) scenarios in refined analyses that aimed to understand the feasibility of screening for psychological distress in a clinical setting [27].

When considering the psychological burden in cancer survivors, an important issue is the possible co-occurrence of multiple psychological symptoms in the same individual since mental health problems are highly comorbid in the general population [28]. We found that in survivors and in the control group, self-reported anxiety and depressive symptoms were significantly associated with self-reported somatic complaints. Likewise, in both groups, somatic symptoms were significantly associated with more severe self-experienced functional impairment. This shared comorbidity pattern may reflect the known association between emotional distress and somatic complaints observed in the general population and in clinical samples [29]. Physical symptoms are frequent in school-aged children, with a reported prevalence between 10–25%, and they are not necessarily related to long-lasting effects [30]. However, according to the present results, physical symptoms may represent a complex phenomenon, leading to perceived functional disability when associated with emotional distress [31]. Prior studies on the mental health/coping outcomes of critically ill young children and their parents showed that parental stress/distress and anxiety about their child’s illness impact the child’s adjustment and coping [32]. In the current study, we explored to what extent emotional, somatic, and behavioral symptoms were reported by young survivors and by their parents relative to healthy peers and their parents, respectively. In the survivors’ group, we found a significant association between the child-reported emotional and somatic symptoms with internalizing behavioral issues and somatic complaints reported by parents. Moreover, in the group of survivors, a positive correlation emerged between children’s and parents’ reports on somatic symptoms and functional impairment descriptions. As a whole, more parents’ concerns were found regarding the emotional and behavioral symptoms of children in the survivors’ group. The abovementioned findings were in favor of a higher awareness of psychological and somatic symptoms among the survivors’ parents compared to the controls. The last result can be considered in the light of the long-lasting disease and hospitalization that was featured in the families of the survivors compared to the controls [33]. Of note, the parent reports of emotional functioning in survivors of childhood ALL were significantly associated with symptoms of parent anxiety [11,12]. As such, parental emotional distress might have directly influenced the perceptions and reporting of the child’s emotional functioning in cancer survivors in the present study. In line with this conclusion, the multivariate analyses showed that cancer-related clinical variables, such as the ages at diagnosis and at ending therapy impacted differentially on the survivors’ scores. In the entire survivors’ group and in the subgroup of survivors with emotional distress, the disease duration had a major effect on the self-reported somatic symptoms and on the children’s functioning. Moreover, younger age at diagnosis was the best predictor for anxiety severity in survivors with emotive distress. Taken together, the multivariate analyses highlighted that some parameters delineating the disease duration had an influence on the occurrence of somatic symptoms in the survivors. Moreover, earlier disease experience may impact the anxiety levels in survivors with emotional distress. These latter findings may be related to the long-term effects of early adverse life-events, such as pediatric cancer. As such, early adversities require significant psychological and neurobiological adaptation, particularly in brain areas involved in fear-response and fear-related learning [34]. In line with the current data, the risk of self-reported hyperactivity problems increased significantly among survivors whose ALL was diagnosed at less than 5 years of age [11].

The present study shows some limitations and it should be viewed in the context of the following considerations. Since pediatric survivors were entirely derived from a single academic center, this sample was not intended to be representative of young cancer survivors in the population. Second, the symptoms were derived from assessment scales filled out by the parents and self-reported questionnaires completed by the participants themselves, and a formal diagnosis of psychiatric disorders was not performed. Finally, many demographic and medical factors were recognized as playing an important role in the psychological outcomes of young cancer survivors [3,8]. In the present study, we found that 16% of pediatric survivors had intelligence levels (total IQ) in the low to borderline average range. When considering all cancer survivors, we did not observe any correlation between IQ scores and psychological symptoms. The contribution of neurocognitive functioning to the psychological outcome of pediatric cancer survivors has recently been examined by a refined strategy based on latent profile analyses [35]. Similarly to the present study, a minority of participants (class 1, 23%) had borderline cognitive levels, while most participants had normal intelligence, with or without an attention deficit. No significant differences were observed across the three classes regarding the children’s self-reported ratings of emotional functioning. However, more parents’ concerns were observed regarding the emotional and behavioral symptoms of children with low to average cognitive functioning [23]. The present study aimed to evaluate the global impact of cancer experience on the development of emotional, behavioral, and somatic symptoms in survivors. The nature of the current data set did not allow for consideration of emotional distress in the parents and other well-established risk factors (e.g., family socioeconomic status, parent education, and other variables related to the family/home environment) that might affect the overall psychological functioning within the context of childhood cancer. Similarly, we were not allowed to search for specific risk-factors among families declining to participate. We did not perceive especially unusual characteristics of these families. To the extent to which pertinent variables were available for non-participants, a selection bias was evaluated. Non-participant data were limited to the children’s age and diagnosis. There were no significant differences in age and diagnosis between the participants and non-participants.

## 5. Conclusions

Despite the considered limitations, the current study included information on psychological symptom profiles in pediatric cancer survivors by considering (a) self- and parent-reported symptoms, (b) an appropriate comparison group, and (c) an almost homogeneous diagnostic sample (childhood ALL). Self-reported symptoms showed similar comorbidity profiles in the distressed survivors and the control peers. The multi-informant evaluation indicated differences in the association of self- and parent-reported symptoms between the survivor and control groups. Survivors’ self-reports of depressive symptoms, somatic symptoms, and functional impairment were significantly correlated with the parent reports of child behavioral concerns, somatic complaints, and functional impairment, respectively. The clinical variables indicating disease duration had influences on the anxiety levels and somatic symptoms perception in survivors. The present study showed that multi-informant assessment is critical for understanding the symptom profiles and for informing interventions with particular regard to parental participation and support.

## Figures and Tables

**Table 1:** Cognitive and psychological symptoms in children survivors of cancer and healthy, age-matched controls.

Clinical Variable	Survivors Mean (SD) (*n* = 40)	Controls Mean (SD) (*n* = 79)	df	*t*	*p* *
Age at study time (years) Cognitive level (WISC III total IQ)	12.9 (3.1) 99.5 (14.74)	12.5 (3.3) 96.15(12.46)	117 79	0.895 0.928	0.256 0.357
Anxiety symptoms (MASC total T-score)	48.30 (10.89)	42.96 (10.22)	116	3.241	**0.0016**
Depression symptoms (CDI total T-score)	46.48 (9.49)	49.08 (7.55)	117	0.606	0.504
Behavioral symptoms (CBCL total T-score)	47.08 (9.82)	43.01 (11.02)	117	2.431	0.016
Somatic symptoms (CSSI-24 child total)	5.56 (7.79)	7.35 (8.94)	112	−0.531	0.597
Somatic symptoms (CSSI-24 parent total)	4.98 (1.91)	5. 23(2.34)	117	0.156	0.876
Functional disability (FDI—child total)	2.58 (3.85)	2.62 (4.60)	112	0.396	0.693
Functional disability( FDI—parent total)	0.44 (1.42)	0.31 (1.23)	117	0.404	0.575

Wechsler Intelligence Scale for Children (WISC) III; Multidimensional Anxiety Scale for Children (MASC); Children’s Depression Inventory (CDI); Children Behavior Check-List (CBCL); Children’s Somatic Symptoms (CSSI-24); Functional Disability Inventory (FDI). * *t*-test. Bonferroni-corrected alpha-level: 0.05/9 = 0.005. The significant *p*-value is in bold.

**Table 2:** Significant correlations between psychological symptoms in young cancer survivors.

Severity Scores	CBCL Total T-Score	Parent CSSI-24 Total	Child CSSI-24 Total	Parent FDI Total	Child FDI Total
MASC total T-score	0.489 * (0.536) ^a^	0.430 ** (0.041)	0.441 ** (0.561) **	0.320 * (0.061)	0.490 ** (0.289)
CDI total T-score	0.735 ** (0.413)	0.559 ** (0.061)	0.554 ** (0.416) **	0.683 ** (0.229)	0.633 ** (0.020)
CBCL total T-score		0.375 * (0.710) **	0.493 * (0.173)	0.430 (0.616) **	0.387 * (−0.364)
Parent-CSSI-24 total			0.350 * (0.199)	0.387 * (0.951) **	
Child CSSI-24 total				0.368 * (0.209) *	0.79 ** (0.801) **
Parent FDI total					0.440 ** (0.237)

^a^ Correlation values in control participants in parenthesis. * *p* < 0.05, ** *p* < 0.01 (uncorrected significance levels). Multidimensional Anxiety Scale for Children (MASC); Children’s Depression Inventory (CDI); Children Behavior Check-List (CBCL); Children’s Somatic Symptoms (CSSI-24); Functional Disability Inventory (FDI).
